# Editorial for the Special Issue “Advanced Energy Storage Materials: Preparation, Characterization and Applications (3rd Edition)”

**DOI:** 10.3390/ma19122561

**Published:** 2026-06-12

**Authors:** Junwei Wu, Chen Liu

**Affiliations:** 1 School of Materials Science and Engineering, Harbin Institute of Technology, Shenzhen 518067, China; 2 Guangdong Provincial Key Laboratory of New Energy Materials Service Safety, Guangdong Research Center for Interfacial Engineering of Functional Materials, College of Materials Science and Engineering, Shenzhen University, Shenzhen 518060, China

## 1. Introduction

The accelerating global transition toward decarbonized energy systems has placed advanced energy storage at the forefront of materials science research [1]. Lithium-ion batteries, sodium-ion batteries, supercapacitors, and emerging solid-state energy storage devices all rely on the rational design of electrode and electrolyte materials whose composition, structure, and interfacial behavior dictate energy density, power density, cycle life, and safety [2,3,4,5]. Despite remarkable progress over the last decade, several persistent gaps continue to constrain further advancement, the relationships between synthesis conditions and microstructural features remain incompletely mapped, the mechanistic origins of long-term capacity fade and impedance growth are still debated, and the integration of laboratory-scale innovations into manufacturable, sustainable cells demands a coordinated treatment of preparation, characterization, and application that has historically been pursued in isolation.

It is at the intersection of these challenges that the third edition of the Special Issue “Advanced Energy Storage Materials: Preparation, Characterization and Applications” was conceived. The eight contributions assembled here, together with the strong readership the Special Issue has attracted (more than 11,000 cumulative views), reflect both the vitality of the field and the appetite of the community for high-quality, mechanistically grounded studies that connect material design to device-level performance [6].

## 2. Contributions of the Special Issue

The eight papers gathered in this Special Issue span a deliberately broad spectrum of material chemistries and methodologies, while sharing a common emphasis on the triad of preparation, characterization, and application. As a group, they illustrate how careful synthetic control, complementary structural and electrochemical analysis, and a clear orientation toward practical performance can be combined to advance the state of the art.

Several contributions concentrate on the rational synthesis of electrode-active materials. They examine how processing parameters, precursor selection, calcination atmosphere, dopant content, and morphology control, propagate through to crystallinity, defect chemistry, and electrochemical response. The structure–property correlations reported in these works extend beyond empirical observation: complementary diffraction, electron microscopy, and spectroscopic measurements are used to identify the specific microstructural features responsible for the observed gains in capacity, rate capability, and cyclability.

Another thread of research tackles the design of carbon-based and composite hosts. The focus lies in manipulating hierarchical structures and surface functionalities to enhance ion transport and accommodate cyclic strain. This line of inquiry is particularly valuable for elucidating how architectural engineering mediates the tug-of-war between high energy and long lifespan, yielding universal design rules applicable to diverse active materials.

A further set of studies turns to electrolyte and interphase engineering, providing structural and spectroscopic evidence linking interfacial composition to long-term cycling stability. By examining the formation, evolution, and failure of solid electrolyte interphases under realistic conditions, these works deepen our mechanistic understanding of capacity fade and offer practical strategies for stabilizing electrode–electrolyte contacts.

These eight papers advance a holistic perspective where preparation, characterization and application converge. This integrated approach, coupled with their commitment to experimental transparency, reflects the evolving standards of modern materials science and promises to steer the direction of future research.

## 3. Future Research Directions

Building on the themes represented in this Special Issue, several directions appear especially promising for the years ahead. First, the development of beyond-lithium chemistries, sodium, potassium, zinc, and multivalent systems will become increasingly strategic as resource and sustainability considerations shape material selection. Second, solid-state architectures and high-safety designs require sustained attention to interfacial stability, mechanical robustness, and scalable processing. Third, operando and in situ characterization, combined with manufacturing-aware synthesis, will be essential to translate laboratory advances into durable, commercially viable cells.

Without energy storage that is both affordable and durable, the promise of renewable grids, electric transport, and resilient infrastructure remains out of reach. It is the bedrock upon which equitable energy access must be built. Advances in storage materials translate, often within only a few years, into measurable improvements in the cost, reliability, and environmental footprint of the energy systems on which billions of people depend. The coming decade is therefore likely to see this discipline assume an even more central role in shaping global energy and industrial development, and material-level innovation will remain the most decisive lever for that transformation.

## 4. Concluding Remarks

It is our hope that the papers collected here will serve not only as a record of recent achievements but also as a stimulus for the next round of research questions. Our sincere thanks go to the authors, reviewers, and the *Materials* Editorial Office for making this issue possible. We trust their work will serve as a cornerstone for future editions.
